# Measurement of PDE5 concentration in human serum: proof-of-concept and validation of methodology in control and prostate cancer patients

**DOI:** 10.1007/s40618-024-02428-w

**Published:** 2024-10-01

**Authors:** Giovanni Luca Gravina, Eugenia Guida, Maria Dri, Renato Massoud, Savino M. Di Stasi, Giorgio Fucci, Andrea Sansone, Susanna Dolci, Emmanuele A. Jannini

**Affiliations:** 1 Department of Experimental Medicine, University of L’Aquila, L’Aquila, Italy; 2 Department of Biomedicine and Prevention, University of Rome Tor Vergata, Rome, Italy; 3 Department of Experimental Medicine, University of Rome Tor Vergata, Rome, Italy; 4 Department of Surgical Sciences, University of Rome Tor Vergata, Rome, Italy; 5 Chair of Endocrinology and Medical Sexology (ENDOSEX), Department of Systems Medicine, University of Rome Tor Vergata, Tower E south, Room E 413, Via Montpellier 1, Rome, 00133 Italy

**Keywords:** Phosphodiesterase type 5, Prostate cancer, Benign prostatic hyperplasia, Novel markers of disease

## Abstract

**Purpose:**

We aimed to investigate if the type 5 phosphodiesterase (PDE5), an enzyme with cardinal biological functions in sexual and cardiovascular health, can be detected and quantited in human serum.

**Methods:**

Blood samples were collected from control male and female subjects. PDE5 levels were measured by a specific ELISA kit. ROC curves weighted for age and serum levels of PSA (male subjects), or age (female subjects) were used to identify the predictive ability in the detection of PCa. Sensitivity, specificity, PPV and NPV values were determined for cut-off value determined during ROC curve analysis.

**Results:**

41 control male subjects, 18 control female subjects, and 55 consecutive subjects, of which 25 were affected by benign prostatic hypertrophy (BPH) and 30 with histologically confirmed prostate cancer (PCa), were studied. PDE5 serum levels were detectable in all subjects (range: 5 to 65 ng/ml). Analysis by MANCOVA identified a significant difference in serum PDE5 between control subjects or hyperplasia patients and PCa patients. Marginal means of serum PDE5 concentrations showed a significant difference (*p* < 0.001). The ROC curve demonstrated that PDE5 serum levels can predict men with or without PCa, with 0.806 AUC value (*p* < 0.0001). Using a 12.705 ng/ml PDE5 serum cut-off yielded sensitivity, specificity, PPV, and NPV of 83.3%, 77.27%, 62.5%, and 91.1% in detecting men with histologically proven PCa, respectively.

**Conclusions:**

We demonstrated, for the first time, that PDE5 levels can be detected in human sera and that PCa patients have significantly higher PDE5 concentration compared to BPH patients or male and female controls. While serum PDE5 level measurement may open new research avenues, the clinical relevance of PDE5 levels in PCa patients deserves further investigation.

## Introduction

The phosphodiesterase (PDE) family includes 11 enzymes (PDE1-PDE11) that regulate cell signaling by hydrolyzing second messengers cyclic adenosine (cAMP) and guanosine (cGMP) monophosphates or both. PDE5, the first cGMP-selective PDE discovered, has been found in several tissues, but it is highly expressed in the male genital tract, mostly in the muscular tunica of penile arteries and vas deferens [1–4]. The role of PDE5 is to hydrolyze the cGMP induced by the gaseous nitric oxide (NO) to the inactive GMP, thus inhibiting the relaxation of the smooth muscle cells (SMCs) promoting vasodilation and erection [5, 6].

Interestingly, in human prostate gland, PDE5 expression has been localized both in the fibromuscular stroma and in the glandular structures [7]. This has been confirmed in rats, where a lobe-specific expression of PDE5 has been identified, with the dorsal lobe expressing lower levels compared to ventral or lateral lobes and with the predominant PDE5 expression site being the acinar wall, and not the blood vessel wall as in other tissues [8]. PDE5 expression has also been identified in several cancer types such as colon adenocarcinoma, esophageal, bladder squamous carcinoma, metastatic breast, pancreatic, glioblastoma, lung, and prostate (PCa) cancers as compared to adjacent normal tissues [4, 9–14] as well as in many cancer cells lines, including PCa cell lines LNCaP and PC3 [15].

We demonstrated that PDE5 is highly expressed in BPH stroma and in about 22% of prostatic adenocarcinomas, with no direct correlation to tumor aggressivity [4]. Considering the frequency of new cases and the death rate for prostate cancer (0.112% and 0.02% per year, respectively, calculated in the interval 2015–2020 [16]) it would be of great interest to identify a molecular marker, apart from PSA, detectable in human serum and positively correlating with PCa.

PDE5 subcellular localization has been shown to map to centrosomes and to the endoplasmic reticulum/Golgi area [2], suggesting that it could be potentially released in the extracellular compartment.

Despite the pivotal role played by PDE5 in several cardiovascular functions and despite its locoregional localization in the tunica media of arterioles, data regarding the possibility to dose this enzyme within the bloodstream are currently not available. Hence, this study aimed to identify the presence of PDE5 in human sera. To this end, we employed a commercially available specific ELISA KIT to assay control subjects and BPH or PCa patients.

## Materials and methods

### Clinical samples

The 55 patients included in our study were recruited at the urology center in Italy (Urological Clinic of the University of Rome Tor Vergata, Italy). Clinical suspect of PCa was based on abnormal PSA and/or suspect digital rectal examination (DRE). According to current guidelines, the serum PSA concentration > 1 ng/mL or > 2 ng/mL at 40 or 60 years, respectively, identified men at increased risk of having clinically significant PCa. Diagnosis of PCa of various Gleason scores was done in 30 according to the European Urological Association guidelines [17], while in 25 subjects the diagnosis, obtained according to the American Urological Association guideline, was benign prostatic hyperplasia (BPH) [18]. Forty-one male subjects attending the clinic for various reasons but resulting negative for both BPH and PCa after clinical, instrumental, and serological diagnosis, and apparently healthy for other endocrine, urological and cardiovascular diseases, were considered as controls. In detail, none of the male participants had serum testosterone below 12.0 nmol/l, the threshold suggestive of hypogonadism, or signs or symptoms usually associated with hypogonadism [19]. To further reduce possible confounding factors, study participants were also asked to abstain from using PDE5 inhibitors (PDE5i) in the two weeks prior to serum collection, although a down-regulation of PDE5 expression might occur only following exposure to a remarkably high dosage of such drugs [20, 21]. Table 1 lists the demographic characteristics of the three subsets of patients. Finally, blood samples from 18 female subjects attending the clinic for various, non-vascular reasons were used as control. This study was approved by the local ethical committee. Written informed consent was obtained from all patients.

**Table 1:** Characteristics of the studied population (males only). BPH: benign prostatic hyperplasia. PCa: prostatic cancer. G: Gleason score. *Bonferroni corrected

Variable	Controls (*N* = 41)	BPH (*N* = 25)	PCa (*N* = 30)	P*
Age, year (mean+/- SD)	63,2+/-13,2	64,7+/-8,9	71,2+/-8,5	0,009
PSA (ng/ml) (median+/- SD)	3,3+/- 1,9	8,3+/- 4,7	10,3+/-5,1	< 0,001
Gleason score			G6: 10 G7: 11 G8: 4 G9: 3 G10: 2	

### PDE 5 and PSA serum measurement

Fresh blood samples were allowed to clot for two hours at room temperature before centrifugation for 20 min at approximately 1000 x g. Serum samples were aliquoted and stored at -20 °C until the analysis. Serum PDE5 was measured using the enzyme-linked immunoadsorbent assay kit (ELISA) according to the manufacturer’s instruction (Cloud-Clone Corp, Houston, TX, USA). The kit reportedly has no significant cross-reactivity or interference between PDE5 and its analogues, and has good coefficients of variation (CV) both for intra-assay and inter-assay (CV < 10% and CV < 12%, respectively). Briefly, 100 µl samples were added per well in triplicate, and after having covered the plate with the special slide, it was incubated for 2 h at 37 °C. After three washes, avidin conjugated to horseradish peroxidase (HRP) was added to each well, and the plate was incubated for 1 h at 37 °C. After a new cycle of washes, TMB substrate solution was added to each well; only wells containing PDE5, biotin-conjugated antibody, and enzyme-conjugated avidin exhibited a change in color. The enzyme-substrate reaction was terminated by adding sulphuric acid solution; the color change was measured using a microplate reader set to 450 nm. Optical density values from the triplicate readings were used to calculate, through interpolation in a standard curve, PDE5 concentrations for each sample.

### Statistical analysis

Because some of the variables we examined were positively skewed and did not satisfy the criteria for normal distribution according to the Shapiro-Wilk test, continuous variables were logarithmically transformed before all statistical tests and then presented as mean and 95% Confidence Interval (CI). Accordingly, continuous variable comparisons were performed by multivariate analysis of covariance (MANCOVA) with age and PSA used as weighting variables. Differences in categorical variables were compared with the chi-square or Fisher’s exact test when appropriate. Pearson correlation was used to measure the associations between PDE5 serum values and PSA, Gleason Score, or age. Receiver operating characteristic (ROC) curves weighted for covariates were generated for PDE5 serum values to determine the predictive ability of this marker in detecting PCa. DeLong’s method for the calculation of the Standard Error of the Area Under the Curve (AUC) was used. Sensitivity, specificity, positive predictive value (PPV), and negative predictive value (NPV) values were determined for a cut-off value determined during ROC curve analysis. The area under a ROC curve represents the probability that a randomly chosen diseased subject is (correctly) rated or ranked with greater suspicion than a randomly chosen non-diseased subject. A *p* value < 0.05 was considered statistically significant.

## Results

Type 5 phosphodiesterase was detected in the sera of all the 114 subjects in this study (30 with histologically confirmed PCa, 25 with BPH, 41 control males, and 18 control females).

**Fig. 1:**
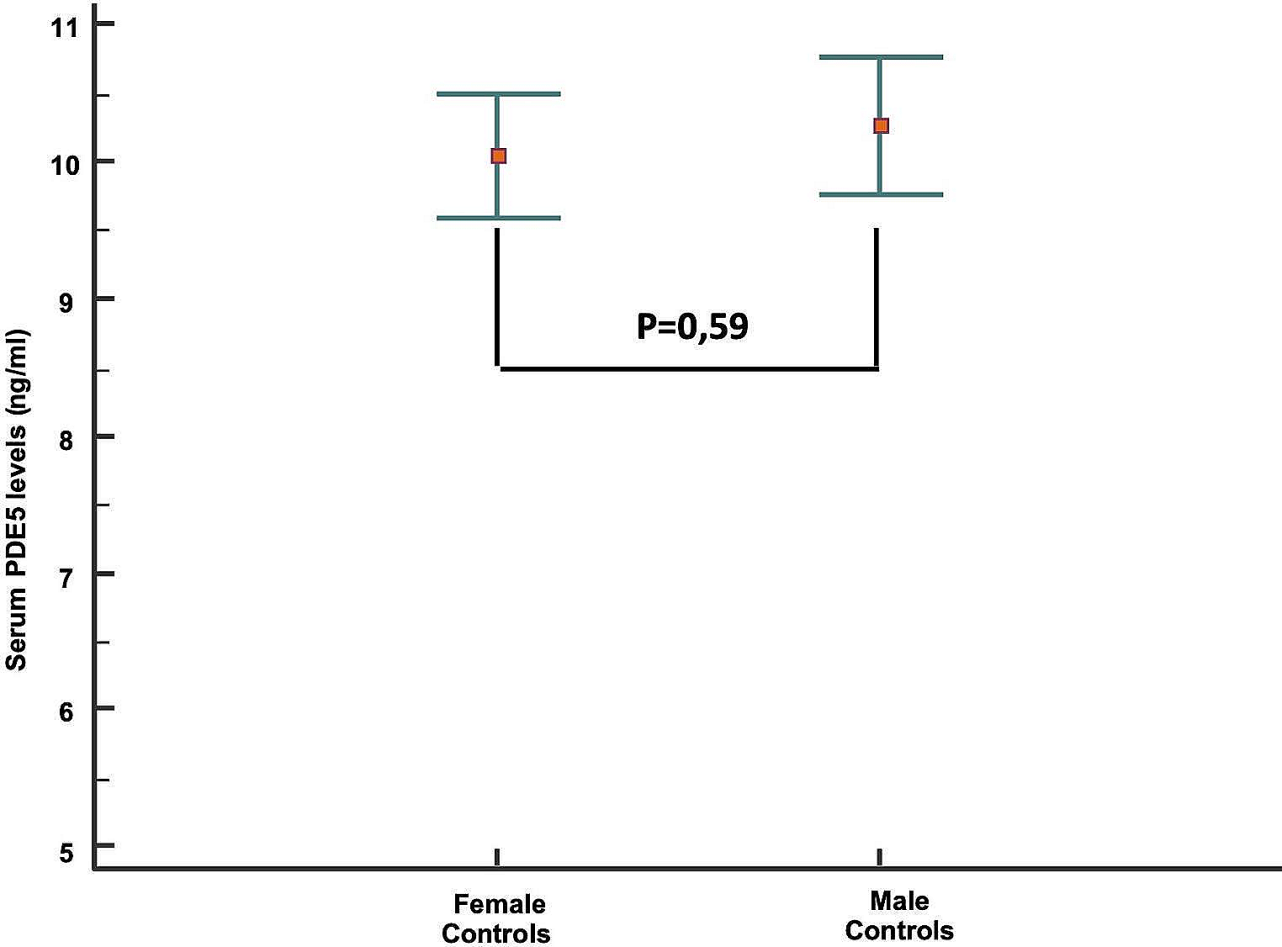
Serum level of PDE5 according to sex between male and female control subjects (M: *n* = 41; F: *n* = 18)

**Fig. 2:**
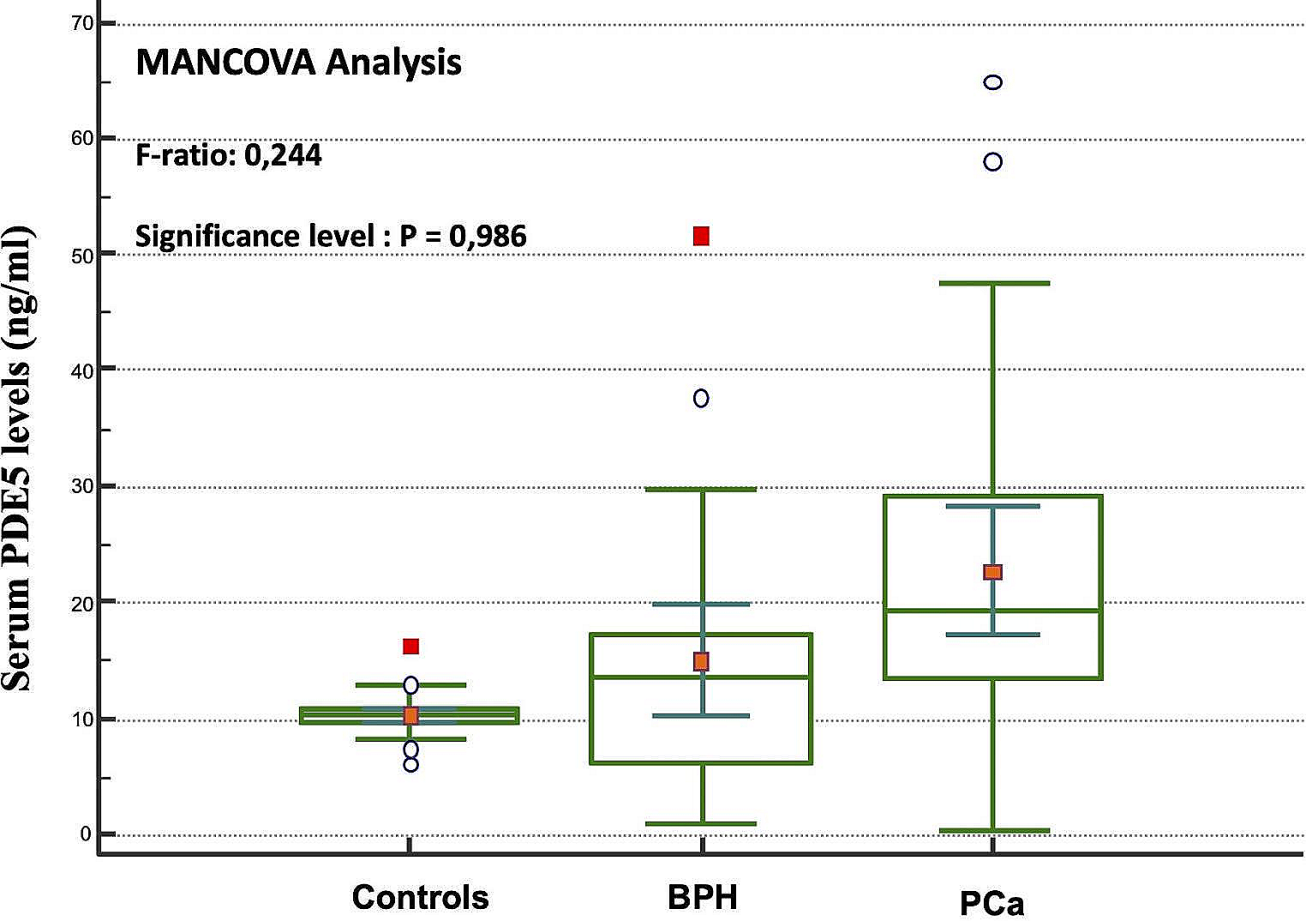
Box-and-whiskers plot of serum PDE5 values in men without urological disease (Controls, *n* = 41), with benign prostatic hyperplasia (BPH, *n* = 25) or with prostate cancer (PCa, *n* = 30)

**Fig. 3:**
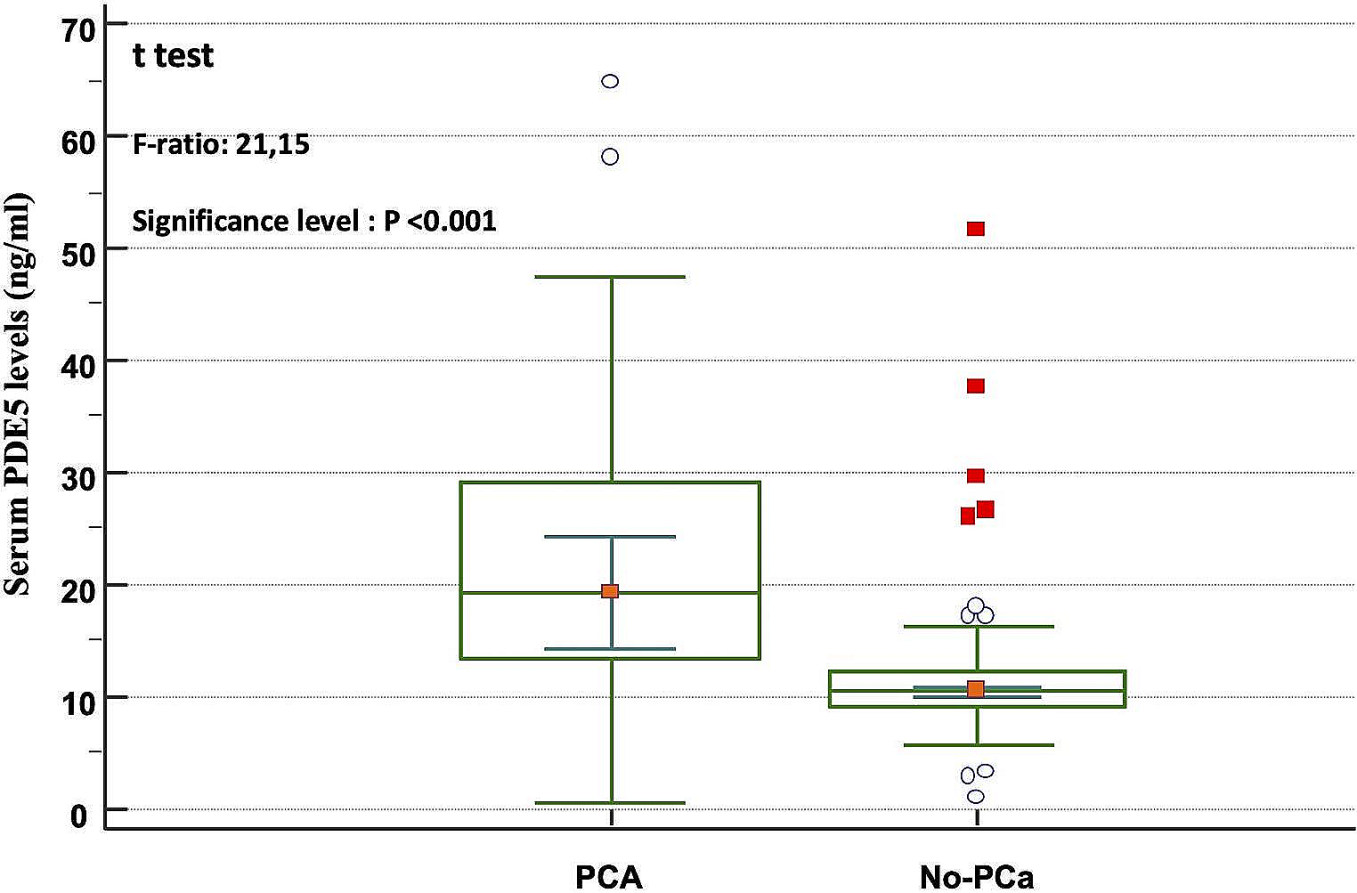
Box-and-whiskers plot of serum PDE5 values in prostate cancer patients (PCa, *n* = 30) and subjects without prostate cancer (No-PCa, *n* = 66)

**Fig. 4:**
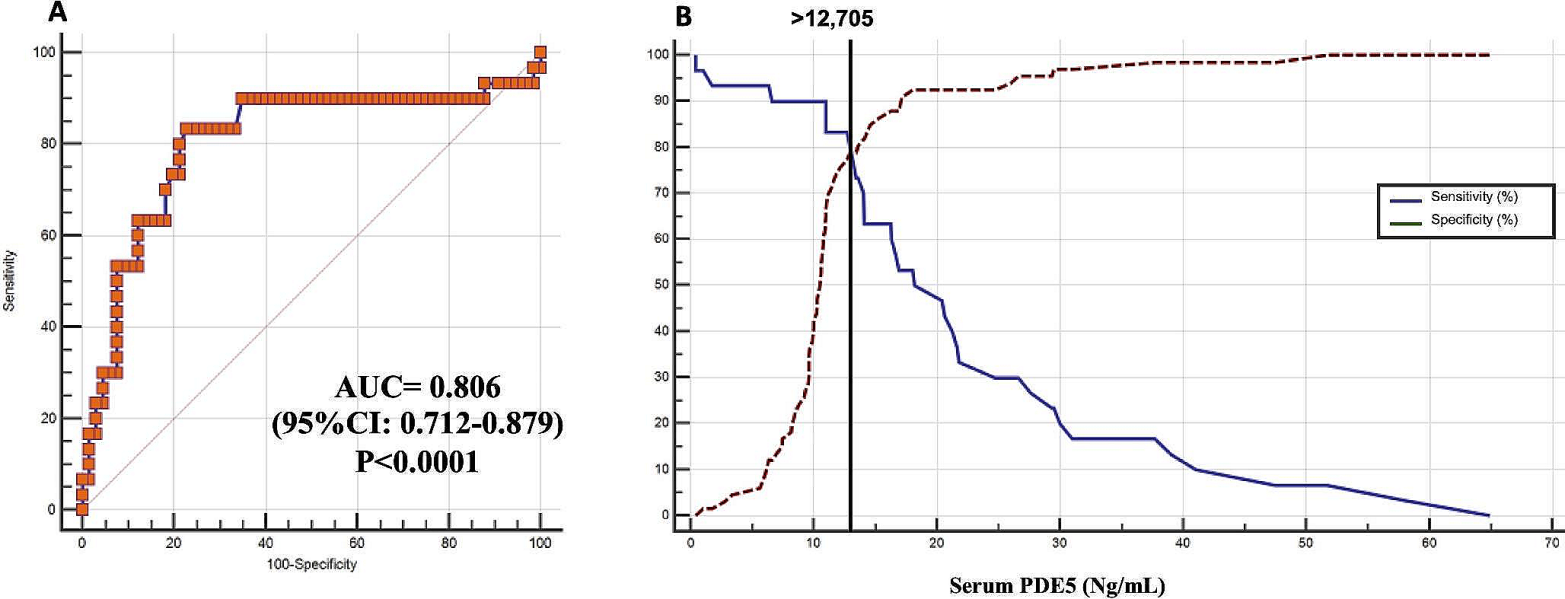
**A:** ROC Curve analysis with AUC value for discriminating men with (*n* = 30) or without (*n* = 66) prostate cancer. **B:** Sensitivity/specificity analysis to identify the optimal cut-off for PCa diagnosis

Serum level of PDE5 was not statistically different according to sex between the control subjects (Fig. 1). On the contrary, in the male subset, the PDE5 serum level increased in subjects with prostatic diseases (Fig. 2): the Scheffé test for all pairwise comparisons showed statistically significantly higher PDE5 levels only for PCa compared to both BPH and male controls. A comparison between the group without malignancy (No-PCa: male controls + BPH group) and that of the group with PCa showed that the latter was characterized by significantly higher serum levels of PDE5 (Fig. 3). Interestingly, no correlation was found between PDE5 serum values and age (*r*=-0.034 CI95% -0.307 to 0.24; *p* = 0.81), serum PSA values (*r* = 0.017; 95%CI -0.26 to 0.29; *p* = 0.9) and Gleason score (-0.04; CI95% -0.41 to 0.34; *p* = 0.84). Finally, the ROC curve analysis weighted for age and serum PSA levels demonstrated a significant diagnostic ability of PDE5 in detecting men with PCa (Fig. 4A), with a 0.806 AUC (95% CI: 0.712–0.879, *p* < 0.0001). The sensitivity analysis showed that for a PDE5 serum cut-off value greater than 12.705 ng/mL an associate sensitivity, specificity, PPV and NPV of 83.3%, 77.27%, 62.5%, and 91.1%, were observed in detecting men with histologically proved PCa (Fig. 4B).

To measure the effects of several potential confounding variables, all statistical tests investigating the difference in the PDE5 serum value among the study groups (PCa, BPH, normal males, normal females) were weighted for age, and the same calculation was performed among males, including serum PSA. When considering female samples, age was the only variable included, while sexual hormones were not evaluated due to the subjects’ postmenopausal age. Since PDE5 serum values were positively skewed in both groups, a logarithmic transformation was used to approximate the distribution of this continuous variable to a normal distribution. The MANCOVA weighted for the above-mentioned variables demonstrated a significant difference between the two groups with respect to PDE5 serum values (Table 2) (*p* < 0.001). Specifically, in the No-PCa group, the mean PDE5 value was 12.01 ± 7.63 ng/ml, whereas in the PCa group, the mean value was 22.63 ± 15.03 ng/ml (95%CI 17.31 to 27.64).

**Table 2:** Results of MANCOVA analysis for serum PDE5 levels between prostate Cancer (PCa) patients and control subjects (No-PCa) weighted on age and total PSA

Tests of Between-Subjects Effects				
Source	Sum of Squares	DF	F	p
Age	5,005	1	0,0461	0,830
Total PSA	121,262	1	1,117	0,293
Coefficient Of Determination R²	0,2196	R²-Adjusted	0,1853	
Estimated Marginal Means				
Groups	Mean	Std. Error	95% Confidence interval	
No-PCa	117,263	13,597	9,0255 to 14,4271	
PCa	232,671	21,460	19,0043 to 27,5298	
Pairwise Comparisons				
Groups	Mean difference	Std. Error	95% CIª	pª
No-PCa Vs PCa	-115,408	27,112	-16,9263 to -6,1553	0,0001

***ªBonferroni corrected***

## Discussion

To date, PDE5 expression studies have been concentrated on identifying its cellular origin and its role within the tissues in which it is expressed, and no attempts have been made to ascertain the presence and role of this enzyme, if any, in systemic blood flow. This work shows for the first time that PDE5 protein levels can be detected and measured in human serum of male and female subjects, either in physiological or pathological conditions, offering the possibility to investigate a potential link between elevated PDE5 levels and disease.

For this purpose, since PDE5 is expressed in the stromal compartment of the prostate [3, 7, 8], and it is targeted by PDE5i [22], we measured and compared its serum levels in benign and malignant neoplastic conditions vs. male controls, finding preliminary pieces of evidence suggesting this protein as a new serum marker to potentially monitor disease occurrence and progression. While we did not find cut-off values that identified a class of subjects or patients among female and male with BPH, we found that PDE5 levels were significantly higher in PCa patients than in the No-PCa group, with a cut-off value of 12.705 ng/ml. We found that men with PCa, irrespectively of the severity of the disease, have higher levels of serum PDE5 when compared to men without this tumoral condition. However, PDE5 serum levels cannot be considered, as PSA, a neoplastic or a tissue marker of prostate disease. In fact, while PSA is a specific marker of the prostatic tissue and mirrors both the dimension and health of this gland, this is not the case for the serum PDE5, which is, on the contrary, largely distributed in several tissues. Moreover, the absence of measurable differences between male and female PDE5 serum levels suggests that steroid hormones do not play a significant role in the modulation of these levels. This hypothesis should be considered cautiously because our subset of women used here as controls were in menopause. However, preliminary data, not shown here for lack of completeness, confirmed also in males a lack of correlation between sexual hormones (androgens) and circulating PDE5 levels. Dedicated studies are needed to ascertain this important point, which was outside our aim.

The diagnostic accuracy of PDE5 measured by ROC curve aligns with the diagnostic accuracy of other well-known markers of malignancy in PCa. In fact, in a large series of 820 subjects undergoing prostate biopsy, the diagnostic accuracy of PSA ratio (F/T PSA) was 0.745, 0.603 for total PSA, 0.618 for PSA density (PSAD), and 0.691 for PSA of transition zone (TZ) [23]. The newer PCA3 urinary test, recently approved for use in men at high risk of PCa, has a ROC curve of 0.678 [24]. In a study of 1.072 subjects scheduled for prostate biopsy, PCA3 presented a ROC AUC of 0.693 for overall cancer detection [25]. More recently, the precursor of PSA ([-2]proPSA) was tested in its ability for cancer detection, showing a ROC curve with an AUC of 0.70 in a population of men with a PSA ranging from 2 to 10 ng/mL [26]. The ROC curve values for other PCa markers are, thus, of the same range as those here found for PDE5 serum levels. A further interesting finding of our study is that a serum PDE5 cut-off greater than 12.705 ng/ml is associated with 83.3% specificity in identifying men with histologically proven PCa. In this regard, the performance of this possible marker in terms of specificity is interesting when considered in the wider context of PCa-specific tumor markers, although our finding should be confirmed in future studies.

We and others have previously demonstrated that PDE5 is highly expressed in the stromal compartment of BPH Sect. [27]. We also found in a low but significant number of PCa samples (22%) that PDE5 is expressed in cancer cells, but not in the surrounding stromal cells, and such expression did not correlate with the tumor aggressiveness, according to their Gleason score [4]. This evidence only partially fits with the results presented here, but it can suggest that the cellular origin of circulating PDE5 could not only be in the tumoral area but also in the neighboring tissue, including tumor-infiltrating immune cells, or even in more distant sites.

So far, the studies on PDE5 have concentrated on the local expression and role in peripheral tissues, and no attempts have been made to ascertain the level and action of this enzyme in the systemic blood flow. The role of serum PDE5 could be different from that in the cell. In fact, the enzyme is localized, in human and experimental animals, in the centrosomes and its intracellular levels are regulated by the mitotic activity of the SMCs, being higher in quiescent, contractile myometrial cultures when proliferation is inhibited [2]. On the contrary, we report here that serum levels of PDE5 are higher in proliferating conditions, such as BPH and PCa. This result could be explained by the release of the enzyme from SMCs and stromal cells of the prostate under inflammatory conditions. However, dedicated experimental protocols are needed to evaluate this speculative hypothesis.

Similarly, monitoring serum PDE5 in PDE5i-treated patients could be helpful in several situations. For example, patients undergoing radical prostatectomy (RP) for PCa are currently treated with PDE5i [28] to restore sexual activity. This treatment has recently raised concerns for biochemical recurrence (BCR) [29]. However, a recent study on 4630 patients ruled out this risk [30] and another study showed that PDE5i may have a protective effect on BCR, as fully acknowledged by the American Urological Association guidelines [31, 32]. Monitoring serum PDE5 levels in such patients will help in understanding if PDE5i can potentially affect PDE5 levels and/or if the PDE5 assay is sensitive enough to reveal any tumor recurrence. Further research are required to provide the necessary evidence.

We previously demonstrated that high levels of PDE5 are immunodetected in quiescent, differentiated myometrial cells [2]; it will then be interesting to monitor PDE5 levels also in patients affected by uterine adenocarcinomas or leiomyomas.

Our findings may have further implications since correlative studies between serum PDE5 levels and erectile dysfunction (ED) or other conditions, such as pulmonary hypertension, may be planned. As our study successfully proved that PDE5 can be measured in human serum, we believe that age-, gender-, and disease- specific threshold for PDE5 measurement could be hypothesized in the near future.

This study has some limitations. Firstly, the relatively low number of studied subjects and the observational nature of the study design may have introduced significant bias. Secondly, as already discussed, we are, at present, unable to demonstrate that the increased PDE5 levels, measured in the sera of men with PCa, are due to the activity of the prostate cancer tissue. Finally, although the three groups differed in the presence or absence of PCa, physiological or unrecognized pathological conditions may have significantly affected the PDE5 production in our subjects. Of note, we believe that the primary outcome of this study, represented by the first proof-of-concept of the measurability of PDE5 in the human sera, was not affected by any of the aforementioned limitations and maintains its importance in novelty and potential clinical application.

## Conclusions

In summary, our study demonstrated, for the first time, that PDE5 is measurable in human sera. Additionally, we provided preliminary evidence that serum PDE5 levels are significantly higher in men suffering from PCa, a promising, novel finding which should be confirmed by further research. The prognostic significance of blood PDE5 levels in benign and malignant prostatic neoplasia remains elusive and will require further investigational assessment.

## References

[1] Carosa E, Rossi S, Giansante N, Gravina GL, Castri A, Dolci S et al (2009) The ontogenetic expression pattern of type 5 phosphodiesterase correlates with androgen receptor expression in rat corpora cavernosa. J Sex Med 6(2):388–396. 10.1111/j.1743-6109.2008.01091.x 19138372 10.1111/j.1743-6109.2008.01091.x

[2] Dolci S, Belmonte A, Santone R, Giorgi M, Pellegrini M, Carosa E, Piccione E, Lenzi A, Jannini EA (2006) Subcellular localization and regulation of type-1 C and type-5 phosphodiesterases. Biochem Biophys Res Commun 341(3):837–846. 10.1016/j.bbrc.2006.01.035 16455054 10.1016/j.bbrc.2006.01.035

[3] Morelli A, Filippi S, Mancina R, Luconi M, Vignozzi L, Marini M et al (2004) Androgens regulate phosphodiesterase type 5 expression and functional activity in corpora cavernosa. Endocrinology 145(5):2253–2263. 10.1210/en.2003-1699 14764637 10.1210/en.2003-1699

[4] Bisegna C, Gravina GL, Pierconti F, Martini M, Larocca L, Rossi P et al (2020) Regulation of PDE5 expression in normal prostate, benign prostatic hyperplasia, and adenocarcinoma. Andrology 8(2):427–433. 10.1111/andr.12695 31433119 10.1111/andr.12695

[5] Yetik-Anacak G, Sorrentino R, Linder AE, Murat N (2015) Gas what: NO is not the only answer to sexual function. Br J Pharmacol 172(6):1434–1454. 10.1111/bph.12700 24661203 10.1111/bph.12700 PMC4369255

[6] Corbin JD, Francis SH (1999) Cyclic GMP phosphodiesterase-5: target of sildenafil. J Biol Chem 274(20):13729–13732. 10.1074/jbc.274.20.13729 10318772 10.1074/jbc.274.20.13729

[7] Uckert S, Oelke M, Stief CG, Andersson KE, Jonas U, Hedlund P (2006) Immunohistochemical distribution of cAMP- and cGMP-phosphodiesterase (PDE) isoenzymes in the human prostate. Eur Urol 49(4):740–745. 10.1016/j.eururo.2005.12.050 16460876 10.1016/j.eururo.2005.12.050

[8] Wang L, Zhang X, Wang G, Visweswariah SS, Lin G, Xin Z, Lue TF, Lin CS (2015) Lobe-specific expression of phosphodiesterase 5 in rat prostate. Urology 85(3):703–e707. 10.1016/j.urology.2014.12.005 10.1016/j.urology.2014.12.005 25733302

[9] Joe AK, Liu H, Xiao D, Soh JW, Pinto JT, Beer DG, Piazza GA, Thompson WJ, Weinstein IB (2003) Exisulind and CP248 induce growth inhibition and apoptosis in human esophageal adenocarcinoma and squamous carcinoma cells. J Exp Ther Oncol 3(2):83–94. 10.1046/j.1359-4117.2003.01076.x 12822514 10.1046/j.1359-4117.2003.01076.x

[10] Piazza GA, Thompson WJ, Pamukcu R, Alila HW, Whitehead CM, Liu L et al (2001) Exisulind, a novel proapoptotic drug, inhibits rat urinary bladder tumorigenesis. Cancer Res 61(10):3961–3968 11358813

[11] Lim JT, Piazza GA, Pamukcu R, Thompson WJ, Weinstein IB (2003) Exisulind and related compounds inhibit expression and function of the androgen receptor in human prostate cancer cells. Clin Cancer Res 9(13):4972–4982 14581372

[12] Whitehead CM, Earle KA, Fetter J, Xu S, Hartman T, Chan DC et al (2003) Exisulind-induced apoptosis in a non-small cell lung cancer orthotopic lung tumor model augments docetaxel treatment and contributes to increased survival. Mol Cancer Ther 2(5):479–488 12748310

[13] Cesarini V, Martini M, Vitiani LR, Gravina GL, Di Agostino S, Graziani G et al (2017) Type 5 phosphodiesterase regulates glioblastoma multiforme aggressiveness and clinical outcome. Oncotarget 8(8):13223–13239. 10.18632/oncotarget.14656 28099939 10.18632/oncotarget.14656 PMC5355091

[14] Huang W, Sundquist J, Sundquist K, Ji J (2020) Phosphodiesterase-5 inhibitors use and risk for mortality and metastases among male patients with colorectal cancer. Nat Commun 11(1):3191. 10.1038/s41467-020-17028-4 32581298 10.1038/s41467-020-17028-4 PMC7314744

[15] Carosa E, Castri A, Forcella C, Sebastiani G, Di Sante S, Gravina GL et al (2014) Platelet-derived growth factor regulation of type-5 phosphodiesterase in human and rat penile smooth muscle cells. J Sex Med 11(7):1675–1684. 10.1111/jsm.12568 24836457 10.1111/jsm.12568

[16] https://seer.cancer.gov/statfacts/html/prost.html

[17] EAU - EANM - ESTRO - ESUR - ISUP - SIOG Guidelines on Prostate Cancer (2024) EAU Guidelines Office. https://uroweb.org/guidelines/prostate-cancer. Accessed April 29 2024

[18] Sandhu JS, Bixler BR, Dahm P, Goueli R, Kirkby E, Stoffel JT, Wilt TJ (2024) Management of lower urinary tract symptoms attributed to Benign Prostatic Hyperplasia (BPH): AUA Guideline Amendment 2023. J Urol 211(1):11–19. 10.1097/JU.0000000000003698 37706750 10.1097/JU.0000000000003698

[19] Isidori AM, Aversa A, Calogero A, Ferlin A, Francavilla S, Lanfranco F et al (2022) Adult- and late-onset male hypogonadism: the clinical practice guidelines of the Italian society of andrology and sexual medicine (SIAMS) and the Italian Society of Endocrinology (SIE). J Endocrinol Investig 45(12):2385–2403. 10.1007/s40618-022-01859-7 36018454 10.1007/s40618-022-01859-7 PMC9415259

[20] Lin C-S, Chow S, Lau A, Tu R, Lue TF (2001) Regulation of human PDE5A2 intronic promoter by cAMP and cGMP: identification of a critical Sp1-Binding site. Biochem Biophys Res Commun 280(3):693–699. 10.1006/bbrc.2000.4221 11162576 10.1006/bbrc.2000.4221

[21] Lin G, Xin Z-C, Lue TF, Lin C-S (2003) Up and down-regulation of Phosphodiesterase-5 as related to Tachyphylaxis and Priapism. J Urol 170(2S). 10.1097/01.ju.0000075500.11519.e8 10.1097/01.ju.0000075500.11519.e8 12853767

[22] Gonzalez RR, Kaplan SA (2006) Tadalafil for the treatment of lower urinary tract symptoms in men with benign prostatic hyperplasia. Expert Opin Drug Metab Toxicol 2(4):609–617. 10.1517/17425255.2.4.609 16859408 10.1517/17425255.2.4.609

[23] Djavan B, Zlotta A, Remzi M, Ghawidel K, Basharkhah A, Schulman CC, Marberger M (2000) Optimal predictors of prostate cancer on repeat prostate biopsy: a prospective study of 1,051 men. J Urol 163(4):1144–1148 discussion 1148–1149 10737484

[24] Marks LS, Fradet Y, Deras IL, Blase A, Mathis J, Aubin SM et al (2007) PCA3 molecular urine assay for prostate cancer in men undergoing repeat biopsy. Urology 69(3):532–535. 10.1016/j.urology.2006.12.014 17382159 10.1016/j.urology.2006.12.014

[25] Aubin SM, Reid J, Sarno MJ, Blase A, Aussie J, Rittenhouse H, Rittmaster R, Andriole GL, Groskopf J (2010) PCA3 molecular urine test for predicting repeat prostate biopsy outcome in populations at risk: validation in the placebo arm of the dutasteride REDUCE trial. J Urol 184(5):1947–1952. 10.1016/j.juro.2010.06.098 20850153 10.1016/j.juro.2010.06.098

[26] Sokoll LJ, Sanda MG, Feng Z, Kagan J, Mizrahi IA, Broyles DL et al (2010) A prospective, multicenter, National Cancer Institute Early Detection Research Network study of [-2]proPSA: improving prostate cancer detection and correlating with cancer aggressiveness. Cancer Epidemiol Biomarkers Prev 19(5):1193–1200. 10.1158/1055-9965.EPI-10-0007 20447916 10.1158/1055-9965.EPI-10-0007 PMC2867076

[27] Zhang W, Zang N, Jiang Y, Chen P, Wang X, Zhang X (2015) Upregulation of phosphodiesterase type 5 in the hyperplastic prostate. Sci Rep 5:17888. 10.1038/srep17888 26657792 10.1038/srep17888 PMC4674741

[28] Zagaja GP, Mhoon DA, Aikens JE, Brendler CB (2000) Sildenafil in the treatment of erectile dysfunction after radical prostatectomy. Urology 56(4):631–634. 10.1016/s0090-4295(00)00659-2 11018620 10.1016/s0090-4295(00)00659-2

[29] Michl U, Molfenter F, Graefen M, Tennstedt P, Ahyai S, Beyer B et al (2015) Use of phosphodiesterase type 5 inhibitors may adversely impact biochemical recurrence after radical prostatectomy. J Urol 193(2):479–483. 10.1016/j.juro.2014.08.111 25196656 10.1016/j.juro.2014.08.111

[30] Flores JM, Vertosick E, Jenkins LC, Cooper J, Benfante N, Sjoberg D et al (2024) Do phosphodiesterase type 5 inhibitors increase the risk of biochemical recurrence after radical prostatectomy? J Urol 211(3):400–406. 10.1097/JU.0000000000003823 38194487 10.1097/JU.0000000000003823 PMC11845976

[31] Burnett AL, Nehra A, Breau RH, Culkin DJ, Faraday MM, Hakim LS et al (2018) Erectile Dysfunction: AUA Guideline. J Urol 200(3):633–641. 10.1016/j.juro.2018.05.004 29746858 10.1016/j.juro.2018.05.004

[32] Danley KT, Tan A, Catalona WJ, Leikin R, Helenowski I, Jovanovic B, Gurley M, Kuzel TM (2022) The association of phosphodiesterase-5 inhibitors with the biochemical recurrence-free and overall survival of patients with prostate cancer following radical prostatectomy. Urol Oncol 40 (2):57 e51-57 e57. 10.1016/j.urolonc.2021.05.031 10.1016/j.urolonc.2021.05.031 34284930

